# Protective Effects of Kaempferol on D-Ribose-Induced Mesangial Cell Injury

**DOI:** 10.1155/2019/7564207

**Published:** 2019-03-31

**Authors:** Ning Zhang, Shaoyang Zhao, Jinni Hong, Weiwei Li, Xuemei Wang

**Affiliations:** Research Studio of Integration of Traditional and Western Medicine, First Hospital of Peking University, Beijing 100032, China

## Abstract

Recently, it has been found that the level of urinary D-ribose in type 2 diabetes is notably higher than that in age-matched normal control, and D-ribose is more reactive in the glycation than D-glucose and induces oxidative stress. Kaempferol is one of the main bioactive components in *Astragalus membranaceus*, with numerous physiological actives, such as antioxidant. The present study investigated the protective effects of kaempferol on D-ribose-treated mesangial cells. CCK-8 and LDH assay were used to test cell viability and cell toxicity. Immunofluorescence and flow cytometry were used to detect the AGE formation and ROS accumulation. GSH level was measured to reflect oxidation resistance. Cell apoptosis was evaluated by Hoechst 33258 staining, AO/EB staining, and western blot. Mitochondrial membrane integrity was detected by JC-1 staining, western blot, and RT-PCR. The change of autophagy level was tested by western blot. The results indicated that D-ribose induced not only cell damage and increased AGE formation and ROS accumulation but also GSH depletion. Further studies demonstrated that D-ribose induced mitochondrial depolarization and the activation of caspase-9/3. But kaempferol could partly block these damages. Subsequently, it was confirmed that kaempferol repaired the autophagy disturbance induced by D-ribose, and 3-MA could reverse the protective effect of kaempferol under D-ribose condition. Our study demonstrated that D-ribose induced AGE accumulation and ROS production in mesangial cell and caused mitochondrial apoptosis, but kaempferol could attenuate these changes and its protective effect might be related to the repair of autophagy.

## 1. Introduction

Within 20-25 years of the onset of diabetes, about 40% of diabetic patients develop into diabetic nephropathy (DN), the most common cause of end-stage renal disease (ESRD) in the world [1]. Nonenzymatic glycation (NEG) is one of the important pathogeneses of DN, and it forms the advanced glycation end products (AGEs). Via interaction with the receptor for AGEs (RAGE), AGEs could activate several intracellular signaling cascades, such as the increase of oxidative stress, and cause aberrant cellular response, like inflammation, apoptosis, and autophagy [2–4].

As a naturally occurring monosaccharide, D-ribose not only is the structural component of DNA and RNA but also is a part of ATP [5]. It was recommended as a supplement of the metabolic therapy for chronic fatigue syndrome [6]. But as reducing sugars, D-ribose is more reactive and rapid in glycation than are other reducing sugars, such as D-glucose [7, 8]. Recently, D-ribose was found notably higher in the urine of type 2 diabetes [9] and administration of D-ribose elevated A*β*-like deposition and tau hyperphosphorylation, which result in cognitive impairment and diabetic encephalopathy [10]. The roles of D-ribose in glycation and its resulting effects in vitro and in vivo have drawn increasing attention [11]. However, it is unknown whether D-ribose induced diabetic nephropathy and mesangial cell damage.

The mesangial cell is mainly contributed to the extracellular matrix (ECM) and is important in the maintenance of the mesangial matrix. It has been demonstrated that AGEs induced mesangial cell dysfunction and apoptosis, and these aberrant cellular responses disturbed glomerular homeostasis and were involved in the pathogenesis of DN [12]. AGEs were confirmed to induce reactive oxygen species (ROS), which played a key role in mitochondrial depolarization-mediated apoptosis, and autophagy may be a protective element in cultured mesangial cells [13–17]. To our knowledge, there is no study that links the effect of D-ribose on autophagy and apoptosis in mesangial cells.

Traditional Chinese medicines are wildly used and characterized with the role of multiple targets in the prevention and treatment of diabetes and its complications [18]. Kaempferol is one of the main components of Chinese traditional medicine *Astragalus membranaceus*, with numerous bioactivities, such as antioxidant, anti-inflammatory, anticancer, antidiabetes, and regulating energy metabolism [19]. Kaempferol was confirmed to decrease the progression of type 2 diabetes [20] and diabetic neuropathic pain [21]. However, there are few studies on how kaempferol works on the treatment of diabetes and its complications.

The present study was designed to test the hypothesis that kaempferol attenuates the mesangial cell damage induced by D-ribose and its relative mechanism. We first confirmed that D-ribose induced AGE accumulation, ROS production, and GSH depletion which subsequently caused the mitochondrial apoptosis. But kaempferol could partly block these results. Then, we performed experiments to study on the role of autophagy in kaempferol's cytoprotective effect and confirmed that autophagy might be a protective factor.

## 2. Material and Method

### 2.1. Material

Kaempferol standard was purchased from the National Institute for the Control of Pharmaceutical and Biological Products (Beijing, China), and the purity of kaempferol was >98%. Cell Counting Kit-8 (CCK-8) was purchased from Dojindo Molecular Technologies (Shanghai, China). Hoechst 33258, AO/EB, and 2′,7′-dichlorofluorescin diacetate (DCFDA) were purchased from Sigma Chemical Co. (St. Louis, MO, USA). LDH assay kit, JC-1 kit, and BCA Protein Kit Assay were from Beyotime Institute of Biotechnology (Nanjing, Jiangsu, China). Fetal bovine serum (FBS), Dulbecco's modified Eagle's medium (DMEM), Ham's F12 medium, and penicillin/streptomycin were from Gibco (CA, USA). The primary antibodies for PARP, caspase-3, caspase-9, Bax, LC3, and HRP-conjugated goat anti-rabbit or anti-mouse were purchased from Cell Signaling Technology (Beverly, MA, USA). The primary antibodies for Bcl-2, p62, and beclin1 were purchased from Santa Cruz Biotechnology (San Diego, CA, USA). The primary antibody for AGEs was from Abcam (MA, USA). Western Chemiluminescent HRP Substrate was purchased from Pierce Scientific (IL, USA). ECL reagent and TRIzol reagent were purchased from Thermo Fisher Scientific (CA, USA).

### 2.2. Cell Culture

Murine glomerular mesangial cells (SV40 MES13) were purchased from the Chinese Academy of Medical Sciences, and cells were cultured in DMEM and Ham's F12 medium (3 : 1) containing 5% FBS and 1% penicillin-streptomycin. Cells were digested with 0.25% trypsin for 3 min and terminated at 37°C in a 5% CO_2_ incubator. All the subsequent procedures were displayed under these conditions: control (5.5 mM glucose), model (30 mM D-ribose), low-concentration kaempferol (30 mM D-ribose with 1 *μ*M kaempferol), medium-concentration kaempferol (30 mM D-ribose with 2 *μ*M kaempferol), and high-concentration kaempferol (30 mM D-ribose with 5 *μ*M kaempferol). All treatments were deprived of FBS.

### 2.3. CCK-8 Assay and LDH Assay

The mesangial cells were inoculated in 96-well plates at a density of 5000/well for 24 h then treated with D-ribose and kaempferol for 48 h as mentioned above. Cell viability was assessed by CCK-8 assay kit, with a 10 *μ*L CCK-8 solution added to each well, and then incubated at 37°C for 1 h. The absorbance was measured with a microplate reader (Bio-Rad Model 550; Bio-Rad Laboratories Inc., Hercules, CA, USA); the test wavelength is 450 nm, and the reference wavelength is 600 nm.

Lactate dehydrogenase (LDH) will rapidly release into the culture supernatant when the cell plasma membrane is damaged. The LDH level in the culture supernatant can be detected to reflect the cell damage. After the cells were treated, the culture supernatant was collected and analyzed with the (LDH) assay kit and the absorbance was detected at 450 nm.

### 2.4. Immunofluorescence Stain

Cells were seeded into 24-well plates at a density of 30000/well for 24 h and treated with D-ribose or kaempferol for 48 h, followed by fixing with 4% paraformaldehyde and punching with 0.25% TritonX-100, and then blocked with 5% BSA for 1 h and incubated with a primary antibody against AGEs (1 : 200) at 4°C overnight. The cells were incubated with Alexa Fluor 488-conjugated secondary antibody for 1 hour at room temperature. Then, the cells were stained with DAPI and observed with a fluorescence microscope.

### 2.5. Measurement of Intracellular Glutathione (GSH)

After being treated with D-ribose or kaempferol for 48 h, mesangial cells were dealt with lysis buffer. Then, the supernatant was collected and the GSH level was measured using a commercial kit (Jiancheng Bioengineering Institute, Nanjing, China).

### 2.6. Cellular Reactive Oxygen Species (ROS) Production Measurement

Mesangial cells were seeded into 6-well plates and treated with D-ribose or kaempferol for 24 h. Then, cells were stained with 10 *μ*M 2′,7′-dichlorofluorescin diacetate (DCF-DA), which is oxidized to fluorescent 2′,7′-dichlorofluorescin (DCF). The intracellular level of ROS was detected by measuring the mean fluorescence intensity by flow cytometry.

### 2.7. Hoechst 33258 Stain

Cells were seeded into 48-well plates at a density of 10000/well and treated with D-ribose or kaempferol as mentioned above for 48 h. After being fixed with 4% paraformaldehyde for 15 min, cells were stained with Hoechst 33258 for 10 min then observed under a fluorescence microscope (IX73, Olympus, Japan).

### 2.8. AO/EB Stain

Cells were seeded into 48-well plates and treated as mentioned above for 48 h, then the mixture of AO/EB (100 *μ*g/mL AO and 100 *μ*g/mL EB mixed in the ratio of 1 : 1) was added at room temperature for 5 min in the dark, and then cells were observed under the fluorescence microscope.

### 2.9. Western Blot Analysis

Cells were lysed by a total protein extraction kit and centrifuged at 10000 × *g* for 10 minutes at 4°C. The protein concentration was determined by a BCA assay kit. A total of 30 *μ*g of protein was loaded on SDS-PAGE and then transferred onto polyvinylidene fluoride (PVDF; Millipore, MA, USA) membranes. The membranes were blocked with 5% bovine serum albumin (BSA) in TBST for 1 hour at room temperature and incubated with the following primary antibodies at 4°C overnight: Bax (1 : 1000), Bcl-2 (1 : 1000), caspase-9 (1 : 1000), caspase-3 (1 : 1000), PARP (1 : 1000), LC3 (1 : 1000), P62 (1 : 200), and beclin1 (1 : 200) in 5% BSA-TBST. Then, the membrane was washed three times for 10 min in TBST before being incubated with horseradish peroxidase-conjugated secondary antibodies (1 : 5000) for 1 hour at room temperature, followed by three times rinses in TBST. Protein bands were visualized by ECL reagents and were quantified with ImageJ software (NIH, Bethesda, MD, USA).

### 2.10. Mitochondrial Membrane Potential Assay

The JC-1 assay kit can be used to assess the mitochondrial depolarization. Cells were seeded on glass coverslips and treated as above, then incubated with JC-1 solution at 37°C for 30 min in the dark and observed with a fluorescence microscope. Cells exhibiting green fluorescence implied they had depolarized mitochondria, and red fluorescence implied normal mitochondria.

### 2.11. RNA Extraction and RT-PCR

TRIzol reagent was used to extract total RNA, and the concentration and purity of RNA were measured by a spectrophotometer (Thermo Fisher Scientific Inc.). 2 *μ*g total RNA was reversed to cDNA according to the instruction of the kit's manufacturer (Thermo, MA, USA). Real-time PCR was performed with SYBR Green PCR kits. Briefly, mix 10 *μ*l SYBR-Green I master mix, 1 *μ*l cDNA, 1 *μ*l primer and 8 *μ*l double-distilled water (ddH2O) together. The RT-PCR thermal cycling protocol was programmed in the CFX96™ Real-Time PCR Detection system (Bio-Rad Laboratories Inc., MA, USA), and conditions were as follows: an initial denaturation step at 95°C for 30 s, followed by 40 cycles of denaturation for 5 s at 95°C, and annealing and extension for 30s at 56°C. As shown in Table 1, the primers are listed.

### 2.12. Statistical Analysis

Dates were expressed as the mean ± SEM for at least three independent experiments. All experiments were repeated at least three times with different cell samples. Comparisons between multiple groups were analyzed by one-way ANOVA followed by the Turkey posttest. *P* < 0.05 was considered to indicate statistical significance.

## 3. Result

### 3.1. Kaempferol Protected Mesangial Cells from Cell Death Induced by D-Ribose

**Figure 1:**
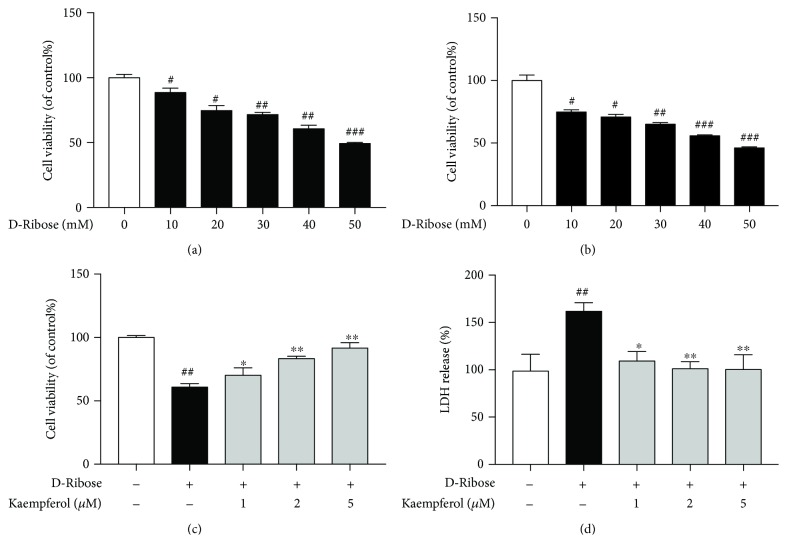
Kaempferol protects mesangial cells from D-ribose-induced cell death. (a) Mesangial cells were exposed to D-ribose (10, 20, 30, 40, and 50 mM) for 24 h. Cell viability was assessed by CCK-8 assay and expressed relative to untreated control cells. (b) Mesangial cells were exposed to D-ribose (10, 20, 30, 40, and 50 mM) for 48 h. Cell viability was assessed by CCK-8 assay and expressed relative to untreated control cells. (c) Mesangial cells were exposed to D-ribose and treated with kaempferol (1, 2, and 5 *μ*M) for 48 h. Cell viability was assessed by CCK-8 assay and expressed relative to untreated control cells. (d) Mesangial cells were exposed to D-ribose and treated with kaempferol (1, 2, and 5 *μ*M) for 48 h. LDH release was expressed as a percentage of the maximum LDH release. Data were expressed as the mean ± SEM from independent experiments. Comparisons between multiple groups were analyzed by one-way ANOVA followed by the Turkey posttest. ^##^*P* < 0.01, relative to the control group; ^∗^*P* < 0.05 and ^∗∗^*P* < 0.01, relative to the D-ribose group.

Mesangial cells were treated with D-ribose (10, 20, 30, 40, or 50 mM) for 24 h or 48 h, and as shown in the results of cell viability, D-ribose induced mesangial cell injury in a dose- and time-dependent manner, and 30 mM D-ribose-treated cells for 48 h could reduce cell viability to about 65%. We chose 30 mM and 48 h as the concentration and duration of the model group, respectively, because it is more like the progression of diabetic nephropathy and it is conductive to follow-up experiments. To evaluate whether kaempferol protected mesangial cells from cell damage induced by D-ribose, we first used CCK-8 assay kit to determine cell viability. As shown in Figure 1(a), the cell viability of the D-ribose group was significantly decreased compared to the control group, which was dose-dependently reversed by kaempferol (1, 2, and 5 *μ*M). Furthermore, this result was confirmed using LDH leakage as a biomarker for cell toxicity (Figure 1(b)). Consistently, D-ribose markedly accelerated LDH release from mesangial cells, which was blocked by kaempferol in a dose-dependent manner. These findings demonstrated that D-ribose induced cell toxicity and decreased cell viability, and it could be attenuated by kaempferol.

### 3.2. Kaempferol Inhibited AGE Formation and Attenuated Oxidative ROS Production Induced by D-Ribose

**Figure 2:**
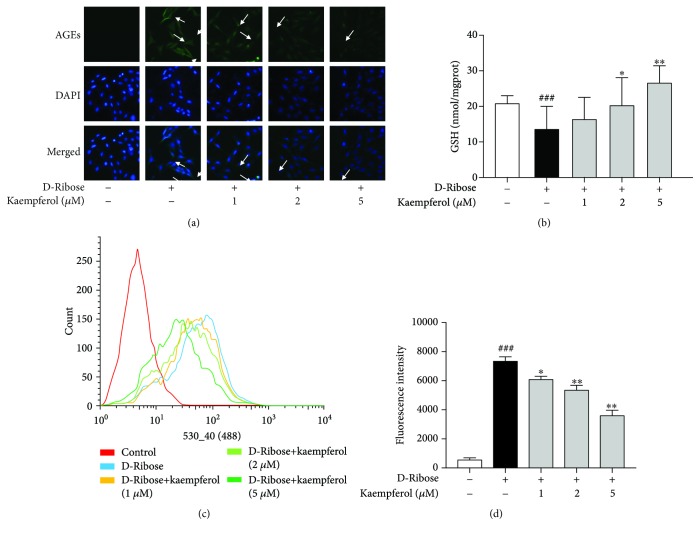
Kaempferol inhibits AGE formation and attenuates ROS production induced by D-ribose. (a) Mesangial cells were treated with kaempferol (1, 2, and 5 *μ*M) for 48 h under D-ribose conditions. Immunocytochemistry assay was used to detect the formation of AGEs, bar = 100 *μ*m. (b) Mesangial cells were treated with kaempferol (1, 2, and 5 *μ*M) for 48 h under D-ribose conditions. The intracellular level of GSH was detected by GSH kit. (c) Mesangial cells were treated with kaempferol (1, 2, and 5 *μ*M) for 24 h under D-ribose conditions. The intracellular level of ROS was detected by measuring the mean fluorescence intensity by flow cytometry. (d) Quantitative analysis of fluorescence intensity. The data are presented as the mean ± S.E.M. from at least three independent experiments. ^##^*P* < 0.01, relative to the control group; ^∗^*P* < 0.05 and ^∗∗^*P* < 0.01, relative to the D-ribose group.

Based on previous studies, D-ribose is more active in glycation than D-glucose is and induces a higher level of advanced glycation end products (AGEs), which could interact with their receptors (RAGE) and subsequently induce oxidative stress. As shown in Figure 2(a), by immunofluorescence staining, we found that D-ribose elevated the formation and accumulation of AGEs significantly in comparison with control, and it could be blocked by the treatment of kaempferol. To further detect whether D-ribose induced oxidative stress, we performed DCF-DA by flow cytometry to assess the production of reactive oxygen species (ROS). GSH is a major naturally occurring antioxidant present in our cells and it can clear intracellular ROS.  Figure 2(b) shows that D-ribose induced GSH depletion and kaempferol could revert it. As depicted in Figures 2(c) and 2(d), kaempferol dose-dependently alleviated ROS production elevated by D-ribose. The results indicated that D-ribose induced AGE accumulation and oxidative stress, and kaempferol partly blocked it.

### 3.3. Kaempferol Attenuated D-Ribose-Induced Mesangial Cell Apoptosis via the Caspase-9/3 Pathway

**Figure 3:**
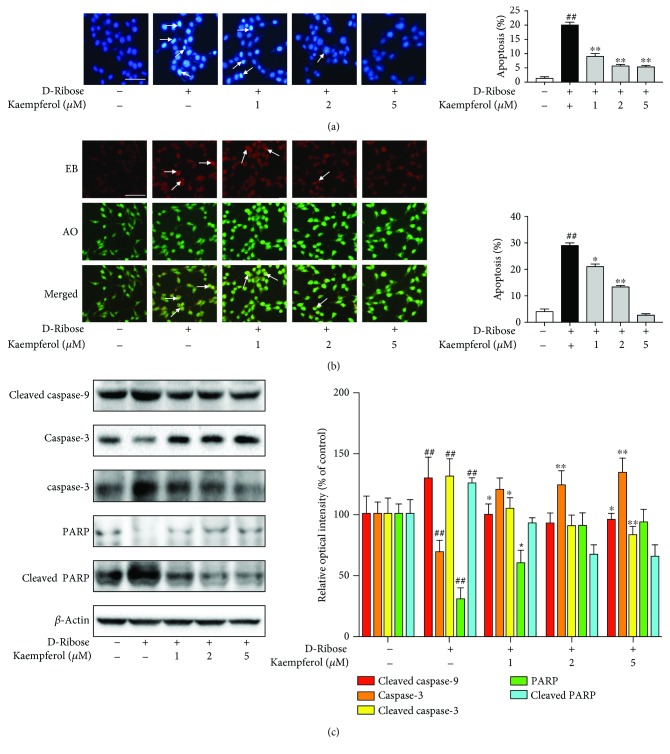
Kaempferol protects mesangial cells against D-ribose-induced apoptosis via the caspase-3/9 pathway. (a-c) Mesangial cells were subjected to D-ribose for 48 h in the presence of kaempferol (1, 2, and 5 *μ*M). (a) Apoptotic nuclei were identified using Hoechst 33258 staining, bar = 100 *μ*m. (b) Apoptotic cells were identified by double staining with acridine orange (AO) and ethidium bromide (EB). Cells which took up both dyes were classified as apoptotic cells, bar = 100 *μ*m. (c) Western blot was used to detect the protein expression of cleaved caspase-9, caspase-3, cleaved caspase-3, PARP, and cleaved PARP. Data were expressed as mean ± SEM from independent experiments. Comparisons between multiple groups were analyzed by one-way ANOVA followed by the Turkey posttest. ^##^*P* < 0.01, relative to the control group; ^∗^*P* < 0.05 and ^∗∗^*P* < 0.01, relative to the D-ribose group.

To further test whether apoptosis played a role in mesangial cells exposed to D-ribose, Hoechst 33258 as one of the DNA dyes was used to detect the cell apoptosis. After staining with Hoechst 33258, a uniform blue fluorescence was shown in the nuclei of healthy cells, while apoptotic cells showed hyperchromatic and dense fluorescent particles within the massive apoptotic nuclei or cytoplasm. As Figure 3(a) shows, there were more hyperchromatic and dense fluorescent particles in mesangial cells treated with D-ribose compared to the control, and kaempferol attenuated the change. These results were further confirmed by acridine orange/ethidium bromide (AO/EB) double stain analysis. AO can enter living and apoptotic cells and emit green fluorescence, but EB only enters apoptotic cells and emits red fluorescence. As depicted in Figure 3(b), D-ribose increased colocalization of EB (red) and AO (green), which was partly blocked by kaempferol. All of these results indicated that D-ribose significantly induced mesangial cell apoptosis, and kaempferol could effectively attenuate the apoptosis. Moreover, to explore the mechanism by which D-ribose induced apoptosis and the role of kaempferol on it, we focus on the caspase-9/3 pathway, an important effect pathway of mitochondrial apoptosis. The results of western blot showed that D-ribose increased the cleaved form of caspase-9/3 and PARP in mesangial cells, and these effects could be reversed by kaempferol (Figure 3(c)). All these indicated that kaempferol effectively protected mesangial cells from D-ribose-induced apoptosis via the mitochondria-dependent caspase-9/3 pathway.

### 3.4. Kaempferol Protected Mitochondrial Membrane Integrity in the Presence of D-Ribose

**Figure 4:**
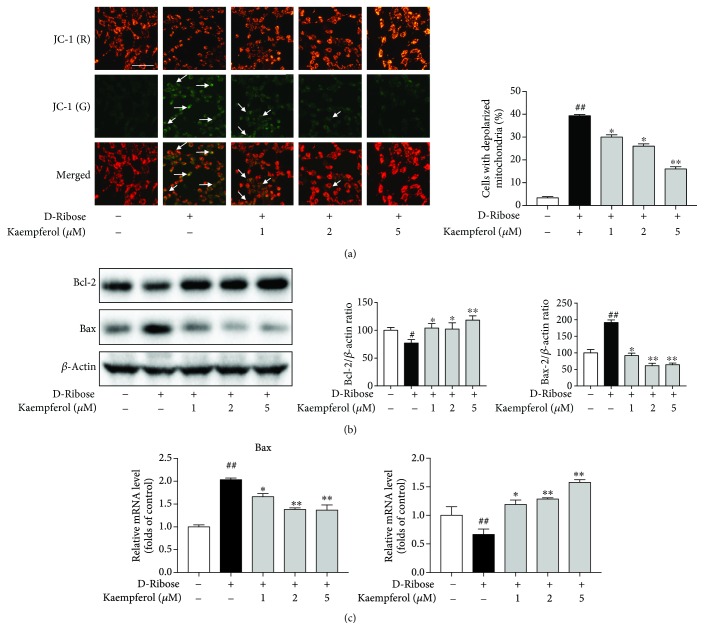
Kaempferol protects mitochondrial membrane integrity. (a-c) Mesangial cells were exposed to D-ribose for 48 h with or without the treatment of kaempferol (1, 2, and 5 *μ*M). (a) JC-1 staining was used to investigate mitochondrial depolarization. Cells with depolarized mitochondria were detected by green fluorescence and quantified as the percentage of total cell, bar = 100 *μ*m. (b) Western blot was used to examine the expression of Bax and Bcl-2 protein. (c) RT-PCR was used to investigate the mRNA level of Bax and Bcl-2. Data were expressed as mean ± SEM from independent experiments. Comparisons between multiple groups were analyzed by one-way ANOVA followed by the Turkey posttest. ^##^*P* < 0.01, relative to the control group; ^∗^*P* < 0.05 and ^∗∗^*P* < 0.01, relative to the D-ribose group.

JC-1 staining is always used to detect the mitochondrial membrane potential, and after staining, the cells with normal mitochondria would emit the red fluorescence, while cells with depolarized mitochondria would emit the green fluorescence. Mitochondrial depolarization can be reflected by the ratio of green fluorescence to red fluorescence. As depicted in Figure 4(a), D-ribose induced a significant increase in cell number with depolarized mitochondria (green fluorescence), and it could be reversed by the treatment of kaempferol in a dose-dependent manner. We further investigated the protein expression of Bax and Bcl-2. Bax is a proapoptosis protein; it can increase the permeability of mitochondria and induce cytochrome C to release and cause cell apoptosis. However, Bcl-2 is an antiapoptosis protein which can maintain mitochondrial membrane integrity. Western blot showed that D-ribose increased Bax protein expression and decreased Bcl-2 protein expression, and these effects could be reversed by kaempferol dose-dependently (Figure 4(b)). Consistently, results of RT-PCR analysis confirmed that D-ribose also elevated the expression of Bax and decrease Bcl-2 at mRNA level, and all these were reversed by kaempferol dose-dependently (Figure 4(c)).

### 3.5. The Protective Effect of Kaempferol in Apoptosis Induced by D-Ribose May Be Related to the Autophagy

**Figure 5:**
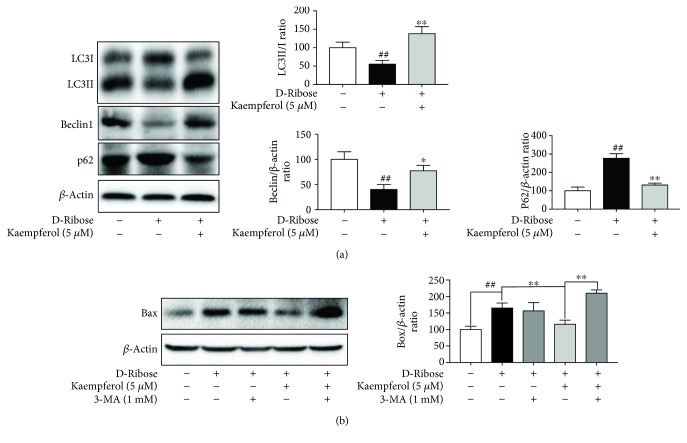
The protective effect of kaempferol in D-ribose-induced apoptosis may be related to the autophagy. (a) Mesangial cells were exposed to D-ribose for 48 h with or without the treatment of kaempferol (5 *μ*M). Western blot was used to investigate the protein expression of LC3, p62, and beclin1. ^##^*P* < 0.01, relative to the control group; ^∗^*P* < 0.05 and ^∗∗^*P* < 0.01, relative to the D-ribose group. (b) Cells were treated with 3-MA (1 mM) and kaempferol (5 *μ*M) for 48 h in D-ribose conditions. The protein level of Bax was determined by western blot, and the protein level of Bax was normalized to the *β*-actin protein level. ^##^*P* < 0.01 and ^∗∗^*P* < 0.01. Data were expressed as mean ± SEM from independent experiments. Comparisons between multiple groups were analyzed by one-way ANOVA followed by the Turkey posttest.

LC3II and beclin1 are well-known biomarkers of autophagosome formation, and p62 is a biomarker of autolysosome degradation. Western blot showed that D-ribose decreased the ratio of LC3II to LC3I and beclin1 was also decreased, but p62 was increased, while treatment with kaempferol (5 *μ*M) could reverse all these effects. This result shows that D-ribose depressed the autophagy, and kaempferol restored the autophagy (Figure 5(a)). To clarify whether the protective effect of kaempferol in apoptosis induced by D-ribose is related to the autophagy, autophagy inhibitor 3-MA was used and Bax was detected. Western blot (Figure 5(b)) showed that there was no significant difference in the expression of Bax in the presence and absence of 3-MA in the D-ribose group. But in the kaempferol treatment group, we found that kaempferol depressed Bax expression induced by D-ribose, which was reversed by autophagy inhibitor 3-MA. These results suggest that the protective effect of kaempferol in D-ribose-induced apoptosis may be related to the repair of autophagy.

## 4. Discussion

As the most common complication of diabetes mellitus, DN has become the leading cause of chronic kidney failure, starting with albuminuria and ultimately leading to end stage renal disease (ESRD) [22]. One of the important mechanisms implicated in the pathogenesis of DN is NEG, which yield AGEs and consequently activate intracellular signaling cascades such as autophagy, apoptosis, and inflammation [23–25]. Studies have demonstrated that the level of AGEs was increased in diabetic patients and diabetic mouse models compared to the control subjects [26, 27]. And attenuation of AGE formation was demonstrated to obviously improve renal function [28].

Mesangial cells play an important role in DN progression; AGEs or other injury factors induce mesangial matrix expansion and mesangial cell apoptosis which are two of the important mechanisms of DN [12, 23].

Chinese traditional medicine has been wildly used in prevention and treatment of diabetes and its complications [18]. *Astragalus membranaceus*, also known as *Huangqi* in Chinese and *Radix Astragali* (RA) in Latin, has been considered as a promising supplement for the treatment of diabetic nephropathy. Kaempferol is the major bioactive constitute of Astragalus membranaceus [19], with a wide range of physiological actives, including antioxidant and antidiabetic [29, 30].

D-Ribose exists in all living cells including the blood and participates in numerous processes [5]. As a reducing sugar, D-ribose has a free carbonyl group which can add to the free amino group of proteins, DNA, and lipoproteins to form AGEs [31, 32], and this process is more active than D-glucose [33]. Recently, an abnormally high level of D-ribose in the urine of type 2 diabetics has been discovered, suggesting that diabetic patients also suffered from D-ribose metabolism disorders [9]. And D-ribose has been regarded as a potential risk factor in type 2 diabetes. There are studies that demonstrated that D-ribose enhanced AGE formation and caused cytotoxicity in different types of cells, such as HEK293T cells, SH-SY5Y cells, and U251 and U87MG astrocytoma cells [8, 34, 35]. In our study, we first demonstrated that D-ribose decreased the cell viability and increased LDH release in mesangial cells, indicating that D-ribose induced the mesangial cell injury. We further detected the AGE formation and confirmed that D-ribose significantly increased the level of AGEs in the mesangial cells. Moreover, we treated mesangial cells with kaempferol, and we found that kaempferol could attenuate the cell injury and AGE formation induced by D-ribose.

High levels of ROS triggered the loss of mitochondrial membrane potential (MMP) which permitted the release of cytochrome c into the cytoplasm and subsequently resulted in the activation of caspase-3 [36]. Studies have demonstrated that treated mesangial cells with AGEs induced cell apoptosis which could be blocked by NAC, a ROS scavenger [14, 23]. GSH plays an important role in cellular antioxidant activity, and it can clear intracellular ROS through nonenzymatic and enzymatic catalysis. Our study showed that D-ribose increased the level of ROS and caused GSH depletion which induced apoptosis via the caspase-3/9 pathway, but all these could be partly blocked by kaempferol. We also detected the mitochondrial depolarization by the JC-1 assay kit; the results indicated that D-ribose caused the loss of MMP and mitochondrial depolarization which also could be partly blocked by kaempferol. We further detected the expression of Bax and Bcl-2, the proapoptotic and antiapoptotic proteins which act on the mitochondrial membrane and influence the MMP. The results showed that D-ribose increased Bax and decreased Bcl-2 at protein and mRNA levels which could be partly blocked by kaempferol. These results consist with the previous study.

Autophagy is an adaptive response under stress conditions which could degrade damaged cell contents through lysosomal hydrolases and maintain cell homeostasis. It has been demonstrated that autophagy could protect cells from proapoptotic insults, and this was likely to be due to the changes in mitochondrial load [37, 38]. And studies demonstrated that autophagy repaired cell damage induced by AGEs and promoted cell survival [14]. Additionally, it has been confirmed that high levels of ROS drive the crosslinking of beclin1, leading to a sequestration of phosphatidylinositol 3-kinase (PI3K) complex 3 and accumulation of p62 [39]. In our study, we found that D-ribose disturbed the autophagy of mesangial cells which could be reversed by kaempferol, indicating that autophagy might play a protective role. To clarify whether the protective effect of kaempferol in apoptosis induced by D-ribose is related to the autophagy, autophagy inhibitor 3-MA was used and Bax was detected. The result showed that the Bax protein levels were not significantly different in the presence and absence of 3-MA in the D-ribose group. But in the kaempferol treatment group, kaempferol depressed Bax expression induced by D-ribose, which was reversed by autophagy inhibitor 3-MA. These results indicated that kaempferol might exert mitochondrial protection and antiapoptosis, and this protective effect might be related with the repair of autophagy under D-ribose injury.

## 5. Conclusions

**Figure 6:**
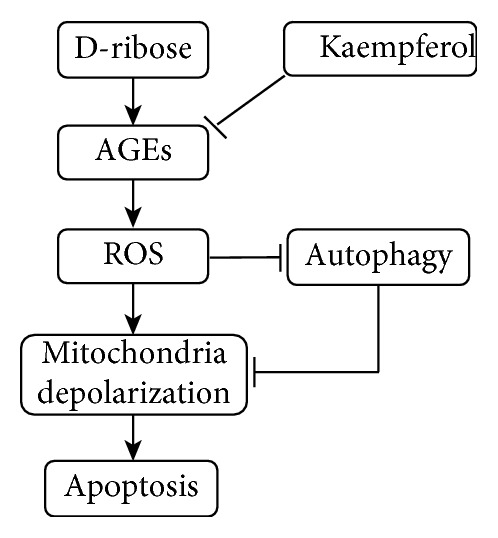
Kaempferol protects the mesangial cell against D-ribose-induced damage.

Taken together (Figure 6), these results confirmed that D-ribose induced AGE formation and ROS production in mesangial cells, which caused the loss of MMP and subsequent activation of the caspase-3/9 pathway and induced cell apoptosis. However, these changes could be partly blocked by kaempferol. Further study indicated that D-ribose caused the disturbance of autophagy, and kaempferol could repair the autophagy which might be related to its cytoprotective effect, but the specific mechanism needs further research to confirm. Our findings elucidated a novel mechanism mediating apoptosis and autophagy induced by D-ribose in mesangial cells, which might help to identify autophagy as an alternative strategy for the therapy of DN.

## Figures and Tables

**Table 1:** Nucleotide sequence of primers used in real-time PCR.

	Forward nucleotide sequence 5′-3′	Reverse nucleotide sequence 5′-3′
*Bax*	AGACAGGGGCCTTTTTGCTAC	AATTCGCCGGAGACACTCG
*Bcl-2*	GCTACCGTCGTGACTTCGC	CCCCACCGAACTCAAAGAAGG
*β-Actin*	GTGACGTTGACATCCGTAAAGA	GCCGGACTCATCGTACTCC

The mRNA levels were normalized to *β*-actin and assessed using the 2^−∆∆Cq^ method.

## Data Availability

No data were used to support this study.
